# Short-Term Neonatal Oral Administration of Oleanolic Acid Protects against Fructose-Induced Oxidative Stress in the Skeletal Muscles of Suckling Rats

**DOI:** 10.3390/molecules24040661

**Published:** 2019-02-13

**Authors:** Trevor Tapiwa Nyakudya, Simon Isaiah, Ademola Ayeleso, Ashwell Rungano Ndhlala, Emmanuel Mukwevho, Kennedy Honey Erlwanger

**Affiliations:** 1 Department of Human Anatomy and Physiology, Faculty of Health Sciences, University of Johannesburg, Doornfontein, Johannesburg 2028, South Africa; trevorn@uj.ac.za; 2 Department of Biochemistry, Faculty of Natural Sciences & Agriculture, North West University, Mafikeng, Mmabatho 2735, South Africa; salmonovic@gmail.com (S.I.); Emmanuel.Mukwevho@nwu.ac.za (E.M.); 3 Department of Biochemistry, Faculty of Science, Adeleke University, P.M.B. 250, Ede 232, Osun State, Nigeria; ademola.ayeleso@gmail.com; 4 Agricultural Research Council, Vegetable and Ornamental Plants (VOP), Private Bag X293, Pretoria 0001, South Africa; 5 School of Physiology, Faculty of Health Sciences, University of the Witwatersrand, Parktown, Johannesburg 2193, South Africa; Kennedy.Erlwanger@wits.ac.za

**Keywords:** oleanolic acid, oxidative damage, neonatal programming, metabolic syndrome, oxidative stress, anti-oxidant enzymes, high fructose

## Abstract

Nutritional manipulations in the neonatal period are associated with the development of negative or positive health outcomes later in life. Excessive fructose consumption has been attributed to the increase in the global prevalence of metabolic syndrome (MetS) and the development of oxidative stress. Oleanolic acid (OA) has anti-diabetic and anti-obesity effects. We investigated the protective potential of orally administering OA in the neonatal period, to prevent fructose-induced oxidative stress, adverse health outcomes and maturation of the gastrointestinal tract (GIT) in suckling rats. Seven-day old Sprague-Dawley rats (N = 30) were gavaged daily with 10 mL/kg of: distilled water (DW), oleanolic acid (OA; 60 mg/kg), high fructose solution (HF; 20% *w*/*v*), or OAHF for 7 days. On day 14, tissue samples were collected to determine clinical health profiles, hepatic lipid content, and activity of anti-oxidant enzymes. Furthermore, biomarkers of oxidative stress and anti-oxidant capacity in the skeletal muscles were assessed. The gastrointestinal tract (GIT) morphometry was measured. Rats in all groups grew over the 7-day treatment period. There were no significant differences in the terminal body masses, GIT morphometry, surrogate markers of general health, liver lipid content across all treatment groups (*p* < 0.05). Neonatal fructose administration decreased the activity of catalase, depleted GSH and increased lipid peroxidation. However, the level of GSH and catalase activity were improved by neonatal OA treatment. Short-term oral OA administration during the critical developmental period protects against fructose-induced oxidative stress without adverse effects on health outcomes associated with MetS or precocious development of the GIT in suckling male and female rats.

## 1. Introduction

Metabolic syndrome (MetS) is a prevalent, multifactorial and complex disease that is associated with a marked increase in the risk to develop metabolic disorders and major cardiovascular consequences [1,2]. According to the global survey data on 195 countries, there are over 600 million obese adults and 100 million obese children [3]. The rise in the global prevalence of MetS has been attributed to the adoption of sedentary lifestyles that are characterised by low physical activity or exercise and the consumption of high-energy diets, especially those that contain fructose [4,5]. The excessive consumption of fructose causes the development of several negative health outcomes associated with metabolic dysfunction such as cardiovascular disease, diabetes mellitus and dyslipidaemia [6,7]. Obesity is regarded as the main causative factor in the development of health outcomes associated with MetS [8]. 

The accumulation of adipose tissue in obesity causes systemic oxidative stress through the production of reactive oxygen species (ROS) from the accumulating adipose tissue [9,10]. The overproduction of ROS by adipocytes contributes to the development of metabolic disorders by decreasing the expression of anti-oxidant enzymes [11]. Oxidative stress, a result of the inability of the anti-oxidant cellular defense mechanisms to reduce ROS, also causes dysregulation of adipocytokines, increases the levels of pro-inflammatory cytokines and oxidative damage by altering mitochondrial bioenergetics [12,13]. Oxidative stress from ROS is also known to cause the development of chronic inflammation by increasing the levels of pro-inflammatory cytokines such as tumour necrosis factor alpha (TNF-α), interleukin-6 (IL-6), and interleukin-1 (IL-1) [14]. Studies performed in adult animal experimental models of metabolic disorders have shown that the activation of cytoprotective anti-oxidant genes can suppress the development of oxidative stress associated with MetS [8,15]. Therapeutic approaches that reduce oxidative stress will, therefore, contribute to the improvement of glycaemic control and prevention of metabolic complications of MetS and T2DM [16]. 

Rats are an altricial species and consequently the pups are born in a relatively underdeveloped state compared to precocial species. Thus, significant post-natal development (which would otherwise occur *in utero* in preococial species) occurs in altricial species. Consequently, the early neonatal period (first couple of days) in rats is comparable to the last gestational trimester of *in utero* human development [17,18,19]. This makes the neonatal period of rat development a critical window of developmental plasticity in which the development of the physiological systems of the pups can be influenced [20]. Animal and epidemiological studies have shown that dietary and pharmacological manipulations during the perinatal suckling period have long-lasting and sometimes irreversible effects in adulthood [21,22,23,24]. This means that developmental programming in rats is not only limited to the *in utero* environment but continues even in the early postnatal period (lactation) where there is continuous growth, rapid development and maturation of various physiological systems [25].

The effect of diet and nutrition, during the neonatal period, on neonatal growth and physiology is important not only because this is a critical stage of developmental plasticity, but also because it potentially has long-lasting positive or negative effects on health in adulthood. Any ingested dietary material contacts the gastrointestinal tract (GIT) first, for digestion and absorption. The GIT is a source of several peptides and hormones that are involved in regulating GIT function and general metabolism [26,27]. Dietary or nutritional manipulations during the neonatal period may therefore cause long-term irreversible positive or negative effects on the development of the GIT and its metabolic function. Some studies in which bioactive phytochemicals in medicinal plant extracts were administered during the suckling period have shown that phytochemicals had a trophic effect on the GIT and caused precocious maturation of the GIT [28]. Due to the limited data from epidemiological and human interventional trials in early life, the role of dietary manipulations on the development of the neonatal GIT using neonatal animal models holds the key to understanding the nutritional interaction during this important developmental period. Due to the rapid growth, development and sensitivity of the GIT during the neonatal period, the neonatal suckling rat is an important experimental model for studying GIT development in early life.

Existing management protocols for the components of MetS involve changes in lifestyle and the use of pharmaceutical and dietary phytochemical agents that target specific biochemical pathways involved in the metabolism of nutrients [29,30]. Phytochemicals have been used alone as nutraceuticals or in combination with standard treatments in the management of MetS [31]. For this study, we selected oleanolic acid (OA), a bioactive triterpenoid phytochemical, due to its proven beneficial pharmacological properties such as anti-diabetic [31,32], hypoglycaemic [33] and anti-oxidant activities [34,35]. OA is also readily available in foodstuffs such as virgin olive oil, fruits and some commonly used medicinal plants [36,37,38]. The anti-diabetic effects of OA observed in adult animal studies has been attributed to its ability to preserve β-cell functionality, improving insulin sensitivity and attenuating fructose-induced hyperglycaemia [39,40]. OA has been shown to exhibit its anti-oxidant properties through enhancing the expression and activity of anti-oxidant enzymes such as glutathione peroxidase (GPx1) and superoxide dismutase (SOD2) [41,42]. An increase in the activity of the anti-oxidant enzymes reduces free radicals and lipid peroxidation [37]. A study conducted in insulin-resistant adult rats showed that OA administration prevented mitochondrial oxidative stress via the activation of nuclear factor (erythroid-derived 2)-like 2-glutamate cysteine ligase (Nrf2-GCLC) signal [37,43]. The anti-oxidant effect of OA is therefore beneficial in the treatment and prevention of metabolic disorders induced by oxidative stress, especially when it is administered in the neonatal period. Previous studies on the beneficial pharmacological effects of OA on MetS have mainly been performed in adult rats and not in suckling rats, especially in the early post-natal period, which is considered as a critical phase of development during which epigenetic changes are likely to cause metabolic changes that exert lifelong effects into adulthood. Moreover, despite the widespread beneficial properties of OA, there seems to be limited knowledge on whether its administration in the neonatal period could protect against oxidative stress, the development of negative health outcomes and precocious maturation of the GIT induced by the administration of fructose in the neonatal period. The current study sought to investigate the potential protective effect of neonatal oral administration of OA against fructose-induced oxidative damage, the development of adverse health outcomes and precocious maturation of the GIT in suckling male and female pups.

## 2. Results

### 2.1. The Effect of Neonatal Oral Administration of Oleanolic Acid on Growth Performance in Suckling Male and Female Rats

**Figure 1:**
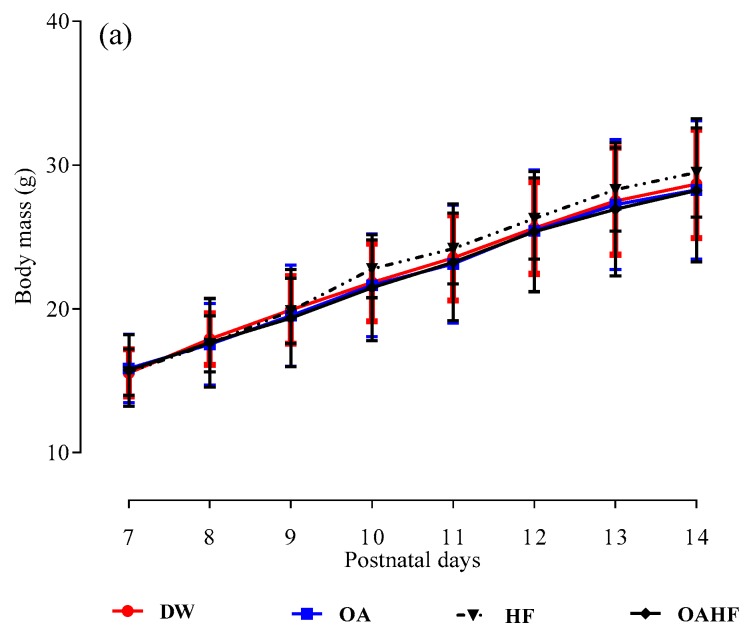

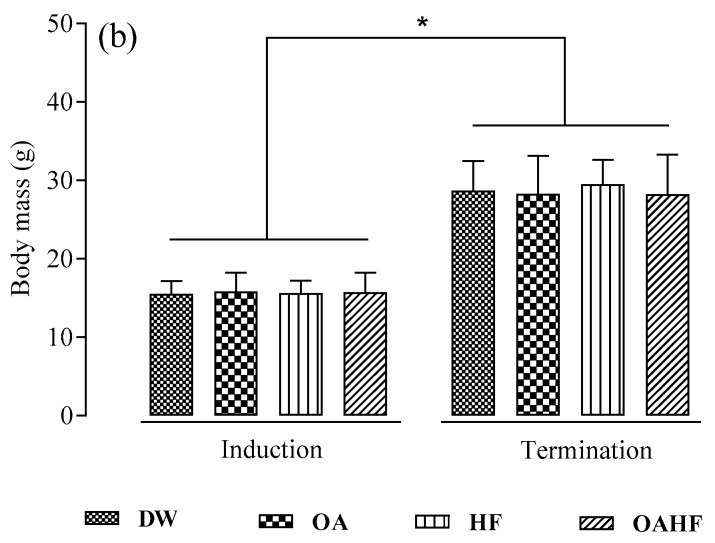
The effect of neonatal oral administration of oleanolic acid on the growth rates (**a**) and the induction (postnatal day 7) and terminal (postnatal day 14) (**b**) of suckling male and female pups. Data presented as mean ± standard deviation. * Significant increase in body mass from induction to termination for all treatment groups (*p* < 0.05). DW = gavaged daily with 10 mL/kg body mass of distilled water with 0.5% (*v*/*v*) dimethyl sulphoxide in the neonatal phase (*n* = 8); OA = gavaged daily with 10 mL/kg body mass of oleanolic acid (60 mg/kg) in the neonatal phase (*n* = 8); HF = gavaged daily with 10 mL/kg of 20% (*w*/*v*) fructose solution in the neonatal phase (*n* = 7); OAHF = gavaged daily with 10 mL/kg body mass of oleanolic acid (60 mg/kg) and 20% (*w*/*v*) fructose solution in the neonatal period (*n* = 7).

The rat pups in all treatment groups exhibited a significant increase (*p* < 0.05; Figure 1a) in body mass over the seven day treatment period (PD7 to PD14). There were no significant differences in the induction and terminal body masses across all the treatment groups (*p* > 0.05; Figure 1b) or in the linear growth parameters such as femoral and tibial bone masses, lengths and bone densities, across the different treatment groups (*p >* 0.05; Table 1).

### 2.2. Effect of Neonatal Oral Administration of Oleanolic Acid on the Gastrointestinal Tract (GIT) and Viscera Organ Morphometry in Suckling Male and Female Pups

There were no significant differences in the lengths and relative masses of the small and large intestines, as well as the absolute and relative masses of the caecum, stomach and kidneys across the different treatment groups (*p* > 0.05; Table 2). 

### 2.3. The Effect of Neonatal Oral Administration of Oleanolic Acid on the General Clinical Health Profiles in Suckling Male and Female Pups

There were no significant differences (*p* > 0.05; Table 3) in the activities/concentrations of surrogate markers of hepatic function (alanine amino transaminase and non-tissue specific alkaline phosphatase), hepatic lipid content, the surrogate markers of renal function (blood urea nitrogen and creatinine). There were also no significant differences in the general clinical biochemistry (phosphate, calcium, total protein, albumin, globulin, glucose and cholesterol) and the concentrations of circulating metabolic substrates (cholesterol and glucose), across all the treatment groups (*p* > 0.05; Table 3).

### 2.4. The Effect of Neonatal Oral Administration of Oleanolic Acid on Anti-Oxidant Enzyme Activity in the Skeletal Muscles of Suckling Male and Female Pups

The administration of high fructose solution in the neonatal period significantly increased the activities of GPx and SOD when compared with the control groups (DW) while treatment of fructose-fed rats with OA did not show significant differences in both GPx and SOD (Table 4). There was a decreased activity of CAT in HF group when compared with the control group (DW). Neonatal treatment with OA prevented fructose-induced decrease in the activity of CAT (*p* < 0.05; Table 4).

### 2.5. The Effect of Neonatal Oral Administration of Oleanolic Acid on Antioxidant Capacity in the Skeletal Muscles of Suckling Male and Female Pups

The anti-oxidant capacity as measured by the Ferric Reducing Antioxidant Power (FRAP) and Trolox Equivalent Antioxidant Capacity (TEAC) is shown in Table 5. 

In this study, there was a significant increase in TEAC value in HF group when compared with the control group (DW) while treatment of fructose-fed rats with OA did not show any significant difference. There were no significant differences in the FRAP values across all groups.

### 2.6. The Effect of Neonatal Oral Administration of Oleanolic Acid on Oxidative Stress Biomarkers in the Skeletal Muscles of Suckling Male and Female Pups

**Figure 2:**
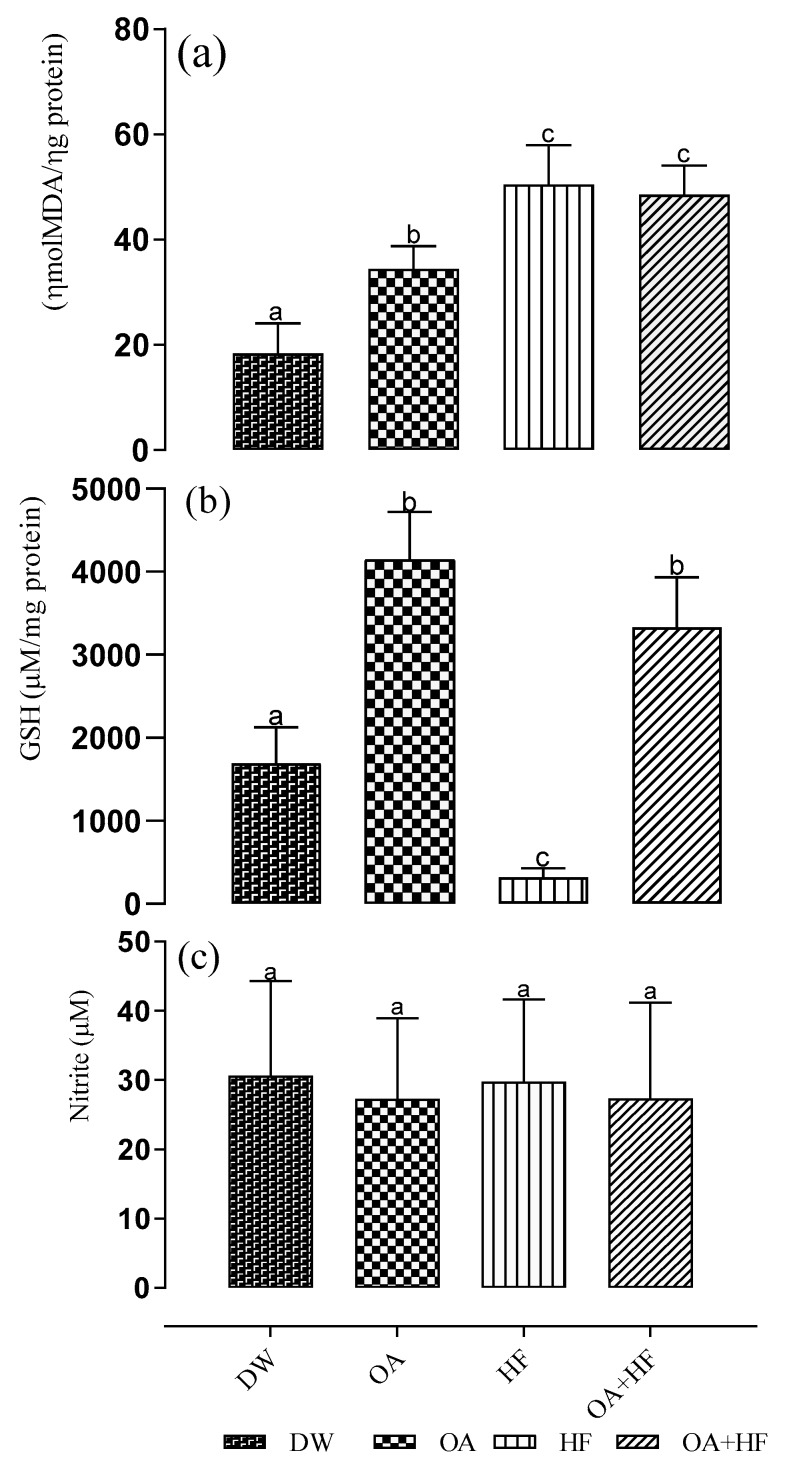
The effect of neonatal oral administration of oleanolic acid on the level of oxidative stress biomarkers in the skeletal muscles of suckling male and female pups (**a**) total glutathione (GSH) level (**b**) and nitrite concentration (**c**) in suckling male and female pups. Data presented as mean ± standard deviation. Bars having same alphabets indicate no significant differences while bars with different alphabets indicate significant differences at *p* < 0.05 across all groups. DW = gavaged daily with 10 mL/kg body mass of distilled water with 0.5% (*v*/*v*) dimethyl sulphoxide in the neonatal phase (*n* = 8); OA = gavaged daily with 10 mL/kg body mass of oleanolic acid (60 mg/kg) in the neonatal phase (*n* = 8); HF = gavaged daily with 10 mL/kg of 20% (*w*/*v*) fructose solution in the neonatal phase (*n* = 7); OAHF = gavaged daily with 10 mL/kg body mass of oleanolic acid (60 mg/kg) and 20% (*w*/*v*) fructose solution in the neonatal period (*n* = 6). Data in the same row with different superscripts is significantly different (*p* < 0.05).

Administration of high fructose solution in the neonatal period resulted in a significant increase in the level of malondialdehyde (MDA) when compared to the control group (*p* < 0.05; Figure 2a). There was no significant difference in lipid peroxidation in the HF group when compared with OAHF. Treatment with OA alone also significantly increased (*p* < 0.05) lipid peroxidation when compared with the control group (DW), but this increase was significantly lower than in the HF groups (*p* < 0.05). The levels of glutathione in high fructose-fed rats were significantly lower in HF group when compared with the other groups (DW, OA and OAHF). (*p* < 0.05; Figure 2b). The decrease in total glutathione levels due to high fructose administration was attenuated by the neonatal administration of OA (*p* < 0.05). There were no significant differences in the nitrite levels across all the treatment groups (*p* > 0.05; Figure 2c). 

## 3. Discussion

This study was designed to investigate the potential protective effect of neonatal (7 days) oral administration oleanolic acid against fructose-induced oxidative stress in the skeletal muscles as well as the development of negative health outcomes and precocious maturation of the GIT in suckling male and female pups. We showed that short-term neonatal administration of OA protected against fructose-induced oxidative stress, had no adverse effects on health and did not cause precocious growth of the GIT in suckling male and female rats.

### 3.1. The Effect of Neonatal Oral Administration of Oleanolic Acid on Growth Performance in Suckling Male and Female Pups

Findings from this study showed that administering OA neonatally via orogastric gavage did not have negative effects on the growth of male and female suckling rats over the 7-day experimental treatment period. Although not statistically significant, high fructose diet (HF) had a moderate growth promoting effect over the same treatment period. Several studies have shown that nutritional [44] and pharmacological [45] manipulation during the early neonatal phase of development has an effect on growth rate and pattern of rats. The growth performance in neonatal animals can have a bearing on physiological systems in adulthood [46,47]. Low birth weight in humans and poor nutrition during the neonatal period affects growth performance and has been associated with the development of chronic illnesses such as hypertension, type 2 diabetes mellitus (T2DM), and obesity later in adult life [48,49].

Body mass changes have previously been used as a measure of growth in rodent studies, but due to fluctuations in factors such as the hydration status of animals and food intake, body mass is deemed to be an unreliable index of growth [50]. Consequently, the use of tibial length as a reliable indicator of linear growth is recommended [51]. Tibiae from rat pups treated with OA and HF had similar lengths compared to the rest of the treatment groups, including the control. This finding further confirms (as shown by the body masses) the non-toxic effects of OA on linear growth in male and female suckling pups. To further assess growth performance, plasma samples could have been used to measure insulin-like growth factor-1 (IGF-1), a hormone that plays an important role in coordinating balanced growth among multiple tissues and organs [52]. However, due to the size of the animals at termination, the volume of blood samples collected were inadequate to perform hormonal assays in addition to the blood for biochemical assays which were undertaken.

### 3.2. The Effect of Neonatal Oral Administration of Oleanolic Acid on the Gross Morphometry of the Abdominal Viscera in Male and Female Suckling Pups

Our results also show that neonatal oral administration of OA did not have any apparent effects on the morphometry of the GIT and accessory structures of the GIT, suggesting that administration of OA does not induce precocious development of the GIT and may not have adverse effects on gut health in neonates. Determination of the morphological characteristics of the developing GIT in neonates has been used as reliable criteria for assessing the effects of dietary treatments on the physiology of neonatal animals [53]. The first port of call for all ingested food is the gastrointestinal tract (GIT), an organ system whose primary function is to digest, produce metabolic regulatory hormones and peptides, extract and absorb nutrients from ingested food among other functions. The GIT is also under direct exposure to the food that we ingest, as such any variations in dietary intake may affect its functionality [54]. Ingested food triggers the release of regulatory hormones and peptides from enteroendocrine cells of the GIT resulting in the modification of GIT function. Unlike the precocious GIT of man which is normally functional at birth, the altricial rat GIT is relatively undeveloped at birth and all of the functional development occurs in the early postnatal period [55]. The GIT of the rat is functionally immature for the first 2 weeks of life, this is followed by rapid development and extensive changes in week 3 [56]. Maturation of the rat GIT occurs by the replacement of cells rather than modification of existing cells and the mucosal mass of the rat becomes fairly constant after about 40 days [57]. Previous studies have shown that the phytochemical consumption during the neonatal period promote the increase in the growth of the GIT [58] and the caecum [28].

As a result dietary changes introduced during suckling, a period of developmental plasticity, could be a potential cause of several diseases, dysfunction of the GIT or positive health outcomes later in adult life [59]. In fact, research has indicated that the alteration in the dietary composition in the early post-natal period has a causal role in metabolic and digestive development in the intestines [60,61,62]. The ingestion of fructose during suckling has been shown to increase body weight and fatty acid uptake into skeletal muscle in adult rats [63].

### 3.3. The Effect of Neonatal Oral Administration of Oleanolic Acid on the General Clinical Health Profiles in Suckling Male and Female Pups

#### 3.3.1. Surrogate Markers of Liver Function

Findings from the current study show that neonatal fructose and OA administration did not affect circulating serum level of the biomarkers of liver function and possibly did not cause adverse hepatocellular changes. Previous studies in adult rats have shown that OA ameliorates hepatic injury and lowers the levels of liver function enzymes after feeding fructose in a dose-dependent manner [64,65]. The liver plays an important role in the metabolism of nutrients such as carbohydrates, lipids and proteins [66,67,68]. The liver also detoxifies harmful chemicals and drugs [68]. Excessive exposure of the liver to dietary and pharmacologic toxic substances may cause hepatocellular damage, particularly the structural integrity of the parenchymal hepatocytes which may affect the hepatic physiology [69]. It is possible that the failure to develop impaired hepatic function following neonatal administration of high fructose solution may be attributed to the absence of GLUT5 fructose transporters whose expression increases post-weaning [70].

In the absence of terminal histology of liver samples, as was the case in this study due to the lack of adequate samples, it is recommended to measure serum or plasma concentration of surrogate biomarkers of liver function for animal experimental research [71]. Surrogate biomarkers of liver function include TP, ALP, AST, ALT and TBIL among others [71]. Total protein gives an estimate of both ALB and GLOB and can also be used to interpret the functional integrity of the liver. Serum ALB indicates the nutritional status and the liver’s synthetic ability, as such any changes in ALB may reflect hepatobiliary irregularities [72].

The commonly measured biomarker of hepatocellular damage which was measured in this study was ALT [73]. ALT is a cytosolic enzyme that is released into the blood after the damage to hepatocytes [74]. Unlike ALP, a non-tissue specific enzyme which is produced by several sources such as bone metabolism and uterus [75], ALT is a reliable measure of the extent of liver damage and the potential hepatotoxicity of pharmaceutical drugs or dietary components [76]. The lack of significant increases in the circulating levels of ALT in suckling rats that received OA suggests that OA had no marked hepatotoxic effects on liver function. Therefore, it is safe to use in the neonatal period.

#### 3.3.2. Renal Function and General Clinical Biochemistry

We also assessed renal function in the pups by measuring the serum concentrations of creatinine, BUN, phosphate, calcium and albumin. Our results showed that short-term neonatal oral administration of OA neither altered renal function nor affected the general clinical health of the suckling male and female pups. Kidneys are important in the homeostatic regulation of body fluids osmolarity, acid-base balance and blood pressure. They also work together with the liver to detoxify and excrete metabolic waste by-products. Creatinine and urea are the most reliable clinical estimates of glomerular filtration rate (GFR), a standard index of renal function [77]. Increased serum concentration of urea and creatinine reflect considerable damage to the kidneys particularly renal tubular function and filtration at the glomerular filtration membrane [78]. The lack of significant changes in plasma urea and creatinine in OA-treated rat pups suggest that neonatal oral administration of OA has no adverse effects on renal function.

#### 3.3.3. Hepatic Lipid Storage and Biomarkers of Metabolic Function

There were no differences observed in hepatic lipid content in male and female suckling pups administered with OA, suggesting that neonatal OA administration had no apparent effects on hepatic lipid content in the suckling rats. In addition to detoxifying xenobiotic substances, the liver plays a major role in the metabolism of various nutrients [79]. The liver is the primary organ in lipogenesis, gluconeogenesis and cholesterol metabolism [80,81]. Metabolic syndrome induces a change in hepatic lipid and carbohydrate metabolism which ultimately causes accumulation and storage of lipids in the liver [79]. This leads to hepatocellular changes associated with non-alcoholic liver disease [82]. The lipids that are stored in the liver come from circulating free fatty acids that are derived from the dysregulation of peripheral lipolysis [80]. The non-fasting plasma glucose and cholesterol levels following the 7-day treatment period were not different across all the treatment groups. This possibly suggests that short-term administration of OA did not negatively affect hepatic glucose and cholesterol metabolism. The rat pups were also not fasted at the time of tissue sampling and this may have affected the findings in our study.

### 3.4. The Effect of Neonatal Oral Administration of Oleanolic Acid on the Activities of Anti-Oxidant Enzymes and Oxidative Stress Biomarkers in the Skeletal Muscles of Suckling Male and Female Pups

We showed that neonatal administration of fructose decreases the activity of CAT, and causes a significant increase in the activities of SOD and GPx in the skeletal muscles. The increase in SOD and GPx activities could be due to more induction of these antioxidant enzymes in helping to scavenge the excessive ROS produced by fructose consumption. The results also showed that OA neonatal supplementation was able to improve the activity of the catalase. It has been reported that fructose causes a significant decrease in the GPx, SOD and CAT activities which in turn accelerates ROS production and potential cellular oxidative oxidative damage [83]. Oxidative stress contributes to the development of metabolic disorders and the damage of macromolecules such as DNA, proteins and lipids ([84]. The activity of anti-oxidant enzymes such as GPx, SOD and CAT is important in the protection of cells against ROS-induced cellular damage [85]. Lipid peroxidation which is caused as a result of the effects of free radicals on lipids having unsaturated fatty acids with more than one double bond could impair the biological membranes’ structure and function in tissues such as skeletal muscles, kidney and liver [86]. The results showed a significant increase in lipid peroxidation (Figure 2a) in the HF group than control, OA and OA + HF treated groups. This indicates that the level of lipid peroxidation; a biomarker of oxidative stress expressed as the degree of accumulation of malondialdehyde (MDA) was significantly increased presumably due to oxidative stress. However, there was no significant effect on lipid peroxidation in the HF group with OA treatment. Although OA treatment alone increased the lipid peroxidation it was less than the impact of HF. The biological significance therefore may be minimal.

The balance between the generation of ROS and the expression/activity antioxidant enzymes is very vital to the maintenance of muscle homeostasis [87]. The investigation also showed a reduction in the concentration of GSH (Figure 2b) in HF group as compared to OA, control and OA + HF groups. Therefore the depletion observed in the HF group was prevented as shown by the significant increase in GSH when treated with OA. Glutathione which reduces oxides, such as hydrogen peroxide, thus protects the DNA from oxidative stress during cell division and growth. In a preclinical study, high fructose diet decrease GSH content in both liver and brain [88]. It can be deduced from this study that the HF diet depleted GSH content in the pups while supplementation with OA increased the GSH content. The nitrite concentrations in all the groups were insignificant.

## 4. Materials and Methods

### 4.1. Ethical Clearance and Study Site

The study was conducted according to the International Standards of Care and Use of Animals in Research, and approved by the Animal Ethics Screening Committee (AESC) of the University of the Witwatersrand, Johannesburg, South Africa (AESC ethical clearance number: 2014/47/D). The in vivo component of the study was conducted in the multi-purpose animal unit of the Central Animal Services at the University of the Witwatersrand, South Africa.

### 4.2. Experimental Animals and Housing

The experiments were performed on the litters of five nursing Sprague Dawley (*Rattus norvegicus*) dams each with between 8–12 (average 10) rat pups, supplied by the Central Animal Services, University of the Witwatersrand. Each dam and its respective litter were housed in the same acrylic cages with stainless steel mesh lids. Wood shavings were used as bedding and changed twice a week. The room temperature was maintained at 25 ± 2 °C. Dams and the rat pups were placed on a 12-h light and dark cycle (with lights on at 07:00 am). There was adequate positive pressure ventilation of the room at all times. The rat pups were marked on their tails with different colour-codes using permanent markers containing non-toxic ink for easy identification. The dams did not receive any experimental treatment but were provided with normal commercial rat chow (Epol^®^, Johannesburg, South Africa) and water ad libitum throughout the suckling period. During the 7-day experimental period, dams were allowed to freely nurse until euthanasia of the rat pups on postnatal day (PD) 14. The dams were also weighed twice a week as part of the routine health monitoring and were returned to stock immediately after euthanasia of their rat pups.

### 4.3. Study Design and Dietary Treatments

The 5-day old rat pups were weighed on the PD5 following parturition and given a day for acclimatization before receiving treatments. On PD6 the rat pups were randomly allocated into four treatment groups each consisting of a minimum of seven mixed male and female rat pups. Rat pups in each litter were assigned to different groups to avoid dam-effect bias. The pups were weighed daily to adjust treatment dosage per body mass and, received the following treatments: Group 1: Control (DW)—distilled water with 0.5% (*v*/*v*) dimethyl sulphoxide which was used as a vehicle control; Group 2: Oleanolic acid (OA)—in which oleanolic acid (60 mg/kg body mass) was administered to investigate the effects of OA alone; Group 3: High fructose solution (HF)—in which rat pups were administered 20% (*w*/*v*) high fructose solution to induce metabolic dysfunction; Group 4: Oleanolic acid and high fructose diet (OAHF)—in which a combination of oleanolic acid (60 mg/kg body mass) and 20% (*w*/*v*) high fructose solution was administered to investigate the protective effects of the OA.

All treatments were administered once daily in the morning (09:00–10:00), for seven days (PD7 to PD13), at a volume of 10 mL/kg body mass via orogastric gavage. After administration of treatments, all the rat pups were monitored for 20 min for unusual behavioural changes and clinical signs of toxicity throughout the course of the experimental treatments of which no adverse events were recorded. Rat pups open their eyes at about 14 days postnatally after which in addition to suckling, they forage on other substances in their vicinity. Thus, the gavaging was restricted to the seven days prior to them opening their eyes.

### 4.4. Terminal Procedures

#### 4.4.1. Sample Collection

At the end of the 7-day experimental treatment period (on PD 14), the pups were euthanised by an intraperitoneal injection of sodium pentobarbital (200 mg/kg body mass; Euthanaze^®^, Centaur Laboratories, Johannesburg, South Africa). For ethical reasons and because of their young age, the rat pups were not fasted prior to termination. The thorax was opened and a 1-mL syringe with a 21-gauge needle was used to collect blood via cardiac puncture. The blood was placed into heparin-coated tubes. The tubes were gently inverted for 30 s to mix the anti-coagulant with the blood and then centrifuged at 3500× *g* at 4 °C for 15 min. The plasma was separated, frozen and stored in microtubes in a freezer at −20 °C for the determination of clinical biochemistry and general health profiles. The triceps muscle samples were dissected out, snap frozen in liquid nitrogen and stored at −80 °C in cryovial tubes until further molecular analyses.

#### 4.4.2. Determination of Visceral Organ Morphometry

Following blood sample collection, the abdomen was cut via a midline incision. The stomach, caeca, liver, kidneys, small and large intestines were carefully dissected out. The luminal contents of stomach, caeca, small and large intestines were emptied by gently squeezing them out after which the gastrointestinal viscera were weighed on a digital analytical balance (Presica 310M^®^; Precision Instruments, Zurich, Switzerland). Gross morphometric measurements of the small and large intestines were determined using a ruler with minimum stretching of the tissues on a dissecting board.

### 4.5. Measurement of Growth Performance

#### 4.5.1. Body Mass Determination

The pups were weighed daily to determine the effects of the different treatments on growth pattern and the adjustment of the dosage of the treatments over the 7-day period.

#### 4.5.2. Determination of Indices of Linear Bone Growth

The left hind leg was detached from each of the carcasses, cleaned off of all the flesh and then femora and tibiae were separated. The bones were dried to constant weight in an oven (Salvis^®^, Salvis Laboratory, Lucerne, Switzerland) at 50 °C for 6 days and then weighed using a balance (Precisa 310M, Precisa Instruments). The lengths of the femora (measured between the distal femoral articular surface to the greater trochanter) and tibiae (measured between tibia head medial malleolus) were measured by a vernier callipers (Hi-impact, Dejuca, South Africa) and were used as indices of linear growth in the pups. Tibial and femoral bone densities (Seedor index) were calculated as follows [89]:

Seedor index (mg/mm) = mass of bone (mg)/length of bone (mm)(1)

### 4.6. Determination of Biochemical Health Profile Markers

The effect of the treatments on the general health status of the rat pups was determined using the plasma samples collected at termination. General biochemical profiles (cholesterol, glucose, phosphate and calcium) surrogate markers of hepatic function (alanine amino transferase, non-tissue specific alkaline phosphatase, albumin and total bilirubin), renal function (blood urea nitrogen and creatinine) and protein profiles (total protein, albumin, and globulin) were measured using a calibrated automatic biochemical analyser (IDEXX VetTest^®^, Clinical Chemistry Analyser, IDEXX Laboratories Inc, Westbrook, ME, USA) as per manufacturer’s instructions. Briefly, stored plasma samples were thawed at room temperature. The samples were then gently inverted to mix the contents and placed into the analyser which automatically drew up 150 µL of the plasma. The analyser then loaded 10 µL of plasma onto each of the 12 pre-loaded disks after which each sample was then analysed and printouts provided. The results from the measurement of enzyme markers were reported as units per litre (U/L).

### 4.7. Determination of Hepatic Lipid Content

The liver samples from each of the four different treatment groups were pooled together and then sent to a South African National Accreditation System-accredited laboratory of the Agricultural Research Council in Pretoria, where the intra-hepatic lipid content was determined. Briefly, the liver samples were freeze-dried and ground into a fine powder which was used for lipid extraction by the Soxhlet method using the Tecator Soxtec System HT 1043 extraction unit (Gemini BV Laboratories, Apeldoorn, The Nederlands). Hepatic lipid content determination was performed in triplicate for each treatment group.

### 4.8. Determination of the Anti-Oxidant Enzyme Activity

Muscular tissue (triceps) samples from −80 °C freezer were collected and kept in ice. They were weighed (gram) and suspended in a 15 mL falcon tube. Homogenate was done in (10% *w*/*v*) freshly prepared phosphate buffer (50 mM Na-Pi, 0.5% (*v*/*v*) Triton x-100, pH 7.5). The samples were homogenized with a homogenizer (Stuart homogenizer SHM1/382, Vernon Hills, IL, USA). Homogenate was centrifuged for 30 min 10,000 rpm at 4 °C (Thermo Scientific SL 8R GO Cat N0. 75007224, Langenselbold, Germany) and supernatant were collected and aliquoted into labelled cryovial tubes and stored in a freezer at −80 °C for further analysis.

#### 4.8.1. Catalase Assay

Catalase activity was determined using the method described by Sinha [90]. A hundred microliters (100 μL) of homogenised muscle sample was measured into labeled Falcon tubes for both test/control test. One hundred microliters (100 μL) of distilled H_2_O was used as standard/blank. To each of control test/blank, 1000 μL distilled H_2_O was added and 1000 μL of hydrogen peroxide (H_2_O_2_ (65 mM) in 50 mmol/L sodium, potassium phosphate buffer) was added to each of samples from the experimental groups and standards. The mixtures were vortexed and incubated at 37 °C for 3 min. One thousand microliters (1000 μL) of dichromate/acetic acid was added to each of the tubes. This was then placed in a water bath and kept at 100 °C for 10 min after which it was left to cool under tap water, then centrifuged to remove precipitated protein (2500× *g* for 5 min). The changes in absorbance were recorded at 570 nm against the reagent blank using spectrophotometry (Thermo Scientific^TM^ Multiskan^TM^ GO Cat N0. N13133/2015 Model, Ratastie 2, FI-01620 Vantaa, Finland). The equation below was used to determine catalase enzyme activity:

Catalase activity of test kU = 2.303/t × [log S^0^/S − M] × Vt/Vs (2)

where: t = time, S^0^ = Absorbance of standard tube, S = Absorbance of test tube, M = Absorbance of control test (correction factor), Vt = Total volume of reagents in test tube and Vs = Volume of plasma.

#### 4.8.2. Superoxide Dismutase Assay

The superoxide dismutase (SOD) activity was determined using SOD assay kits-WST (Sigma-Aldrich^®^, St. Louis, MO, USA) following manufacturer’s instructions. Twenty microliters (20 μL) of sample homogenate was measured into each of sample and blank 2 well, and 20 μL of distilled water (H_2_O) was measured into blank 1 and blank 3 well. Two hundred microliters (200 μL) of WST working solution was added to each well and it was mixed. Twenty microliters (20 μL) of dilution buffer was added to blank 2 and blank 3 and 20 μL of enzyme working solution was added to both sample and blank 1. These mixtures were then shaken thoroughly to mix. The microplate reader (Thermo Scientific^TM^ Multiskan^TM^ GO) was incubated at 37 °C for 2 min and the absorbance was recorded at 450 nm. The equation below was used to determine the SOD activity:

SOD (inhibition rate %) = {[(Ablank 1 − Ablank 3) − (Asample − Ablank 2)]/(Ablank 1 − Ablank 3)} × 100(3)

#### 4.8.3. Glutathione Peroxidase and Glutathione Assay

Glutathione peroxidase activity was determined using the method described by Roctruck et al. [91]. Standard working solution of glutathione (GSH) was prepared (0.0307 g GSH dissolved in 100 mL (0.2 M) EDTA solution (pH 8)) in 25 μM, 50 μM, 100 μM, 150 μM, and 250 μM concentration. To 500 μL of phosphate buffer (K_2_HPO_4_ and KH_2_PO_4_; 100 mM (pH 7.4)) in falcon tubes, 100 μL of sodium azide (NaNO_3_; 10 mM) and μL hydrogen peroxide (H_2_O_2_; 2.5 mM) were added. Five hundred microliters of homogenised sample from the different treatment groups was added; followed by the addition of 600 μL of distilled H_2_O. The reaction mixture was incubated at 37 °C for 3 min after which 500 μL of Trichloro acetic acid (TCA, 10% 2 g of TCA dissolved in 20 mL H_2_O) was added and thereafter centrifuged at 3000× *g* for 5 min. To 100 μL of each of the supernatants/standards, 200 μL of K_2_HPO_4_ and 100 μL of DTNB (5’-5’-dithiobis-(2-dinitrobenzoic acid) was added and the absorbance was read at 412 nm with the use of spectrophotometry (Thermo Scientific^TM^ Multiskan^TM^ GO).

Glutathione peroxidase activity was observed by plotting the standard curve and the concentration of the remaining GSH was extrapolated from the curve:

GSH consumed = 245.34 − GSH remaining(4)

Glutathione peroxidase = GSH consumed/mg protein(5)

Results are expressed as µM/mg protein.

### 4.9. Determination of Anti-Oxidant Capacity Assay

#### 4.9.1. Ferric Reducing Anti-Oxidant Power (FRAP)

The FRAP assay was performed using the method described by Benzie and Strain [92]. The FRAP reagent was a mixture of 30 mL acetate buffer (pH 3.4), 3 mL tripyridyltriazine (TPTZ), 3 mL FeCl_3_ and 6.6 mL distilled water (dH_2_O). The homogenised samples (100 µL) from the treatment groups were mixed with 200 µL FRAP reagent in a micro plate (Brand Puregrade Ref: 781600 plates, Brand GMBH + CO KG, Wertheim, Germany). The mixture was incubated for 30 min at room temperature and the absorbance was read at wavelength of 539 nm using a spectrophotometer (Thermo Scientific^TM^ Multiskan^TM^ GO). Ascorbic acid (AA) was used as the standard and the results were expressed as μmolAAE/mL sample.

#### 4.9.2. Trolox Equivalent Anti-Oxidant Capacity (TEAC)

TEAC assay was analysed using the principle of 2,2-azino-bis 3-ethylbenzothiazoline-6-sulphonic acid (ABTS) radical scavenging activity as described by Ou, et al. [93]. The ABTS was prepared by mixing 88 μL K_2_S_2_O_8_ and 5 mL ABTS and left overnight before use. The sample or standard (25 µL) was mixed with 200 µL ABTS solution and incubated for 30 min at room temperature. The absorbance of the mixture was then read at 734 nm using spectrophotometer (Thermo Scientific^TM^ Multiskan^TM^ GO). Trolox was used as the standard and the results were expressed as μmol TE/mL.

### 4.10. Determination of Oxidative Stress Biomarkers

#### 4.10.1. Lipid Peroxidation Assay

Tissue malondialdehyde (MDA) concentration were measured spectrophotometrically as thiobarbituric reactive substance by the method of Buege and Aust [94]. Briefly aliquot mixtures of 20 µL sample or standard (MDA) were mixed with 100 µL of 15% (*w*/*v*) trichloroacetic acid and 100 µL of 0.375% (*w*/*v*) thiobarbituric acid. This mixture was heated at 100 °C for 15 min and the cooled to room temperature then centrifuged at 3000× *g* for 5 min and absorbance measured at 535 nm. Results were expressed as nmolMDA/ng protein.

#### 4.10.2. Nitrite Assay

Nitrite concentration was measured by the Griess reaction method [95]. Briefly, 100 µL of Griess reagent (1% sulfanilamide, 0.1% naphthylethylenediamide in 5% phosphoric acid) was mixed with 50 µL of homogenised samples and incubated for 10 min at room temperature, and absorbance was read at 540 nm using a microplate reader. The result was expressed as µM.

#### 4.11. Statistical Analysis

Data were expressed as mean ± standard deviation (SD) and analysed using GraphPad Prism for Windows Version 7.0 (GraphPad Software Inc., San Diego, CA, USA). Samples from male and female rats were pooled together for all the variables that were measured as there were no significant sex differences across all treatment groups. A two-way repeated measures analysis of variance (ANOVA), with Bonferroni post-hoc test, was used to analyse body mass changes with day as a within-subjects factor and treatment as a between-subjects factor. A one-way ANOVA with Bonferroni post-hoc test was used to compare the means for all the other parameters measured. The level of significance acceptable was *p* ≤ 0.05.

## 5. Conclusions

The findings from this study provide evidence that short-term neonatal oral administration of OA protects against fructose-induced oxidative damage with seemingly no adverse effects on health or the maturational and developmental changes of the gastrointestinal tract in suckling male and female pups. The prophylactic use of OA in the fight against metabolic syndrome during the critical developmental period does not seem to carry health risks. The long term impact of the interventions with OA need to be investigated epigenetically as well as using precocial species which better represent the situation in higher order animals.

## Figures and Tables

**Table 1:** The effect of neonatal oral administration of oleanolic acid on tibial and femoral masses, lengths and Seedor indices in suckling male and female pups.

Parameter	DW	OA	HF	OAHF
**Tibia**				
Mass (mg)	38.50 ± 1.4	35.9 ± 2.0	37.6 ± 1.1	34 ± 2.8
Length (mm)	15.2 ± 0.7	15 ± 0.8	17.1 ± 0.9	14.8 ± 0.9
^¥^ Seedor index (mg/mm)	2.5 ± 0.1	2.4 ± 0.1	2.2 ± 0.1	2.30 ± 0.1
**Femur**				
Mass (mg)	37.8 ± 5.8	33.5 ± 4.5	37.6 ± 8.5	34.1 ± 5.5
Length (mm)	11.4 ± 0.9	11.6 ± 0.7	11.9 ± 1.7	11.1 ± 1.5
Seedor index (mg/mm)	3.3 ± 0.5	2.9 ± 0.3	3.2 ± 0.4	3.1 ± 0.4

Data presented as mean ± standard deviation. DW = gavaged daily with 10 mL/kg body mass of distilled water with 0.5% (*v*/*v*) dimethyl sulphoxide in the neonatal phase (*n* = 8); OA = gavaged daily with 10 mL/kg body mass of oleanolic acid (60 mg/kg) in the neonatal phase (*n* = 8); HF = gavaged daily with 10 mL/kg of 20% (*w*/*v*) fructose solution in the neonatal phase (*n* = 7); OAHF = gavaged daily with 10 mL/kg body mass of oleanolic acid (60 mg/kg) and 20% (*w*/*v*) fructose solution in the neonatal period (*n* = 7). ^¥^ Seedor index = bone density in mg/mm.

**Table 2:** The effect of neonatal oral administration of oleanolic acid on the lengths, absolute and relative weights of visceral organs in suckling male and female pups.

Parameter	DW	OA	HF	OAHF
SI (cm)	54.44 ± 9.23	46.75 ± 16.48	52.24 ± 5.06	53.34 ± 8.59
SI (g)	0.74 ± 0.19	0.74 ± 0.22	0.84 ± 0.27	0.93 ± 0.32
SI ^¥^ rTL	0.49 ± 0.14	0.45 ± 0.15	0.49 ± 0.14	0.62 ± 0.19
LI (cm)	7.09 ± 0.79	6.63 ± 2.72	7.64 ± 0.56	7.47 ± 0.55
LI (g)	0.12 ± 0.01	0.13 ± 0.02	0.13 ± 0.02	0.13 ± 0.03
LI rTL	0.08 ± 0.01	0.08 ± 0.02	0.08 ± 0.00	0.09 ± 0.01
Liver (g)	0.90 ± 0.17	0.94 ± 0.17	0.94 ± 0.16	1.01 ± 0.21
Liver rTL	0.60 ± 0.13	0.63 ± 0.13	0.55 ± 0.10	0.68 ± 0.13
Caecum (g)	0.08 ± 0.02	0.06 ± 0.03	0.07 ± 0.01	0.07 ± 0.02
Caecum rTL	0.04 ± 0.02	0.04 ± 0.02	0.04 ± 0.00	0.05 ± 0.01
Stomach (g)	0.21 ± 0.02	0.21 ± 0.05	0.20 ± 0.03	0.20 ± 0.01
Stomach rTL	0.14 ± 0.02	0.14 ± 0.03	0.12 ± 0.02	0.13 ± 0.01
Kidneys (g)	0.38 ± 0.04	0.27 ± 0.04	0.38 ± 0.03	0.32 ± 0.14
Kidneys rTL	0.25 ± 0.03	0.27 ± 0.04	0.23 ± 0.03	0.22 ± 0.10

Data presented as mean ± standard deviation. DW = gavaged daily with 10 mL/kg body mass of distilled water with 0.5% (*v*/*v*) dimethyl sulphoxide in the neonatal phase (*n* = 8); OA = gavaged daily with 10 mL/kg body mass of oleanolic acid (60 mg/kg) in the neonatal phase (*n* = 8); HF = gavaged daily with 10 mL/kg of 20% (*w*/*v*) fructose solution in the neonatal phase (*n* = 7); OAHF = gavaged daily with 10 mL/kg body mass of oleanolic acid (60 mg/kg) and 20% (*w*/*v*) fructose solution in the neonatal period (*n* = 7); LI = large intestine; SI = small intestine; ^¥^ rTL = organ masses expressed relative to tibial length (g/cm).

**Table 3:** The effects of neonatal oral administration of oleanolic acid on biomarkers of renal and hepatic function, hepatic lipid storage, general clinical biochemistry and the concentrations of circulating metabolic substrates in suckling male and female pups.

Parameter	DW	OA	HF	OAHF
BUN (mmol/L)	4.6 ± 0.7	4.2 ± 0.9	5.2 ± 0.6	4.3 ± 0.5
CREA (µmol/L)	16.9 ± 3.2	18 ± 0	10.3 ± 3.4	14.4 ± 7.1
TBIL (µmol/L)	4 ± 2.7	5.5 ± 3.3	8 ± 3.8	4.12 ± 2.1
PHOS (mmol/L)	2.8 ± 0.2	3.17 ± 0.5	2.9 ± 0.3	3.2 ± 0.3
CAL (mmol/L)	2.7 ± 0.4	2.0 ± 0.9	2.3 ± 0.6	2.5 ± 0.7
TPROT (g/L)	38.1 ± 4.1	41.3 ± 3.7	41.4 ± 5.2	40 ± 3.0
ALB (g/L)	21.1 ± 2.9	19.5 ± 2.4	20.17 ± 2.7	19.3 ± 1.6
GLOB (g/L)	17 ± 4.5	21.7 ± 2.0	21.3 ± 1.2	20.4 ± 2.9
ALT (U/L)	35 ± 5.9	45 ± 14.1	51.8 ± 15.5	43.9 ± 7.2
ALP (U/L)	299.1 ± 63.8	309.3 ± 58.5	394.3 ± 70.3	269.4 ± 62.7
* Hepatic lipid content (%)	2.8 ± 0.0	2.7 ± 0.0	3.2 ± 0.0	3.2 ± 0.0
Glucose (mmol/L)	7.8 ± 1.3	7.6 ± 1.0	8.1 ± 0.3	8.5 ± 1.7
Cholesterol (mmol/L)	4.3 ± 0.4	4.3 ± 0.5	4.3 ± 0.5	4.4 ± 0.8

Data presented as mean ± standard deviation. DW = gavaged daily with 10 mL/kg body mass of distilled water with 0.5% (*v*/*v*) dimethyl sulphoxide in the neonatal phase (*n* = 8); OA = gavaged daily with 10 mL/kg body mass of oleanolic acid (60 mg/kg) in the neonatal phase (*n* = 8); HF = gavaged daily with 10 mL/kg of 20% (*w*/*v*) fructose solution in the neonatal phase (*n* = 7); OAHF = gavaged daily with 10 mL/kg body mass of oleanolic acid (60 mg/kg) and 20% (*w*/*v*) fructose solution in the neonatal period (*n* = 7). BUN = blood urea nitrogen; TBIL = total bilirubin; ALB = albumin; CREA = creatinine; PHOS = phosphate; CAL = calcium; TPROT = total protein; GLOB = globulin; ALT = alanine amino transaminase; ALP = alkaline phosphatase; * Hepatic lipid content expressed as a percentage of liver mass.

**Table 4:** The effects of neonatal oral administration of oleanolic acid on the activities of antioxidant enzymes in the skeletal muscles of suckling male and female pups.

Parameter	DW	OA	HF	OAHF
GPx (µM/mg protein)	308.5 ± 19.4 ^a^	627.5 ± 45.7 ^b^	555.3 ± 29.3 ^b^	533.2 ± 31.1 ^b^
SOD activity (% tissue inhibition rate)	78.2 ± 3.6 ^a^	93.4 ± 0.9 ^b^	96.6 ± 0.4 ^b^	94.1 ± 1.2 ^b^
CAT activity (kU/L) in tissue	1.1 ± 0.0 ^a^	1.4 ± 0.1 ^b^	0.5 ± 0.0 ^c^	1.0 ± 0.0 ^ad^

Data presented as mean ± standard deviation. Identical letters indicate no significant differences while different letters indicate significant differences at *p* < 0.05 across all groups. DW = gavaged daily with 10 mL/kg body mass of distilled water with 0.5% (*v*/*v*) dimethyl sulphoxide in the neonatal phase (*n* = 8); OA = gavaged daily with 10 mL/kg body mass of oleanolic acid (60 mg/kg) in the neonatal phase (*n* = 8); HF = gavaged daily with 10 mL/kg of 20% (*w*/*v*) fructose solution in the neonatal phase (*n* = 7); OAHF = gavaged daily with 10 mL/kg body mass of oleanolic acid (60 mg/kg) and 20% (*w*/*v*) fructose solution in the neonatal period (*n* = 6). GPx = Glutathione peroxidase, SOD = Superoxide dismutase, CAT = Catalase. Data in the same row with different superscripts is significantly different (*p* < 0.05).

**Table 5:** The effects of neonatal oral administration of oleanolic acid on the antioxidant capacity in the skeletal muscles of suckling male and female pups.

	DW	OA	HF	OAHF
TEAC µM/mL)	356.4 ± 13.4 ^a^	392.8 ± 5.79 ^a^	469.3 ± 11.9 ^b^	393.9 ± 12.4 ^a^
FRAP (µM/mL)	36.98 ± 5.24 ^a^	30.16 ± 2.54 ^a^	30.22 ± 1.68 ^a^	36.95 ± 4.56 ^a^

Data presented as mean ± standard deviation. Similar letters indicate no significant differences while different letters indicate significant differences at *p* < 0.05 across all groups. DW = gavaged daily with 10 mL/kg body mass of distilled water with 0.5% (*v*/*v*) dimethyl sulphoxide in the neonatal phase (*n* = 8); OA = gavaged daily with 10 mL/kg body mass of oleanolic acid (60 mg/kg) in the neonatal phase (*n* = 8); HF = gavaged daily with 10 mL/kg of 20% (*w*/*v*) fructose solution in the neonatal phase (*n* = 7); OAHF = gavaged daily with 10 mL/kg body mass of oleanolic acid (60 mg/kg) and 20% (*w*/*v*) fructose solution in the neonatal period (*n* = 6). TEAC = Trolox equivalent anti-oxidant capacity; FRAP = Ferric reducing anti-oxidant power. Data in the same row with different superscripts is significantly different (*p* < 0.05).
